# Diabetes-related lower extremity complications in a multi-ethnic Asian population: a 10 year observational study in Singapore

**DOI:** 10.1007/s00125-021-05441-3

**Published:** 2021-04-22

**Authors:** Tessa Riandini, Deanette Pang, Matthias P. H. S. Toh, Chuen Seng Tan, Daveon Y. K. Liu, Andrew M. T. L. Choong, Sadhana Chandrasekar, E Shyong Tai, Kelvin B. Tan, Kavita Venkataraman

**Affiliations:** 1 Saw Swee Hock School of Public Health, National University of Singapore and National University Health System, Singapore, Republic of Singapore; 2 Policy Research & Evaluation Division, Ministry of Health, Singapore, Republic of Singapore; 3 National Public Health and Epidemiology Unit, National Centre for Infectious Diseases, Singapore, Republic of Singapore; 4 Information Management, Group Health Informatics, National Healthcare Group, Singapore, Republic of Singapore; 5 Department of Surgery, Yong Loo Lin School of Medicine, National University of Singapore, Singapore, Republic of Singapore; 6 Division of Vascular and Endovascular Surgery, National University Heart Centre, Singapore, Republic of Singapore; 7 Department of Vascular Surgery, Tan Tock Seng Hospital, Singapore, Republic of Singapore; 8 Department of Medicine, Yong Loo Lin School of Medicine, National University of Singapore, Singapore, Republic of Singapore

**Keywords:** Amputation, Diabetes-related lower extremity complications, Epidemiology, Incidence rate, Progression, Risk factors

## Abstract

**Aims/hypothesis:**

Diabetes progression and complication risk are different in Asian people compared with those of European ancestry. In this study, we sought to understand the epidemiology of diabetes-related lower extremity complications (DRLECs: symptomatic peripheral arterial disease, ulceration, infection, gangrene) and amputations in a multi-ethnic Asian population.

**Methods:**

This was a retrospective observational study using data obtained from one of three integrated public healthcare clusters in Singapore. The population consisted of individuals with incident type 2 diabetes who were of Chinese, Malay, Indian or Other ethnicity. We examined incidence, time to event and risk factors of DRLECs and amputation.

**Results:**

Between 2007 and 2017, of the 156,593 individuals with incident type 2 diabetes, 20,744 developed a DRLEC, of whom 1208 underwent amputation. Age- and sex-standardised incidence of first DRLEC and first amputation was 28.29/1000 person-years of diabetes and 8.18/1000 person-years of DRLEC, respectively. Incidence of both was highest in individuals of Malay ethnicity (DRLEC, 36.09/1000 person-years of diabetes; amputation, 12.96/1000 person-years of DRLEC). Median time from diabetes diagnosis in the public healthcare system to first DRLEC was 30.5 months for those without subsequent amputation and 10.9 months for those with subsequent amputation. Median time from DRLEC to first amputation was 2.3 months. Older age (*p* < 0.001), male sex (*p* < 0.001), Malay ethnicity (*p* < 0.001), Indian ethnicity (*p* = 0.014), chronic comorbidities (nephropathy [*p* < 0.001], heart disease [*p* < 0.001], stroke [*p* < 0.001], retinopathy [*p* < 0.001], neuropathy [*p* < 0.001]), poorer or missing HbA_1c_ (*p* < 0.001), lower (*p* < 0.001) or missing (*p* = 0.002) eGFR, greater or missing BMI (*p* < 0.001), missing LDL-cholesterol (*p* < 0.001) at diagnosis, and ever-smoking (*p* < 0.001) were associated with higher hazard of DRLEC. Retinopathy (*p* < 0.001), peripheral vascular disease (*p* < 0.001), poorer HbA_1c_ (*p* < 0.001), higher (*p* = 0.009) or missing (*p* < 0.001) LDL-cholesterol and missing BMI (*p* = 0.008) were associated with higher hazard of amputation in those with DRLEC. Indian ethnicity (*p* = 0.007) was associated with significantly lower hazard of amputation.

**Conclusions/interpretation:**

This study has revealed important ethnic differences in risk of diabetes-related lower limb complications, with Malays most likely to progress to DRLEC. Greater research efforts are needed to understand the aetiopathological and sociocultural processes that contribute to the higher risk of lower extremity complications among these ethnic groups.

**Graphical abstract:**

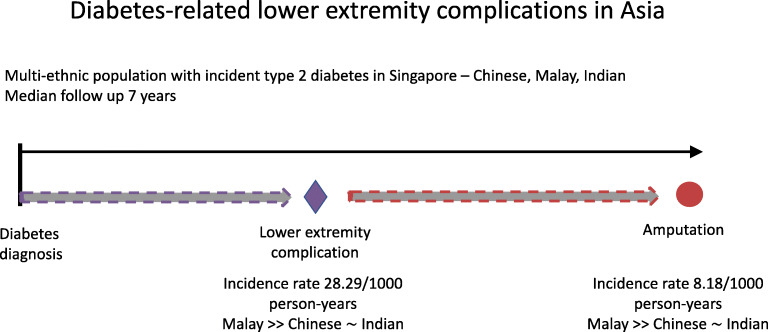

**Supplementary Information:**

The online version contains peer-reviewed but unedited supplementary material available at 10.1007/s00125-021-05441-3.

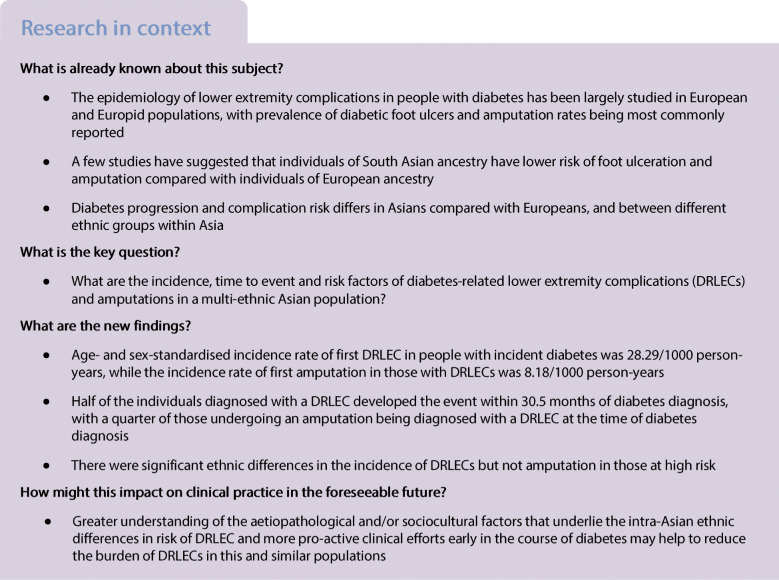



## Introduction

Type 2 diabetes is one of the major public health problems in the world. The growing diabetes epidemic is of major concern especially in Asia, as the continent is home to the largest number of people with diabetes [1]. Lower extremity complications, including peripheral neuropathy, peripheral arterial disease, ulceration, infection, gangrene and amputations, contribute substantially to the disability burden, loss of quality of life, cost of care and mortality in individuals with diabetes [2–5]. These complications, especially the more serious sequalae, are largely preventable with greater care support and integration [6, 7]; however, the burden from diabetes-related lower extremity complications (DRLECs) has been increasing globally [2].

Epidemiological data on DRLECs most commonly report incidence estimates of lower extremity amputations (hereinafter referred to as amputations) and prevalence estimates of diabetic foot ulcers, where substantial inter-country variations have been reported [8, 9]. Differences in clinical protocols, referral pathways and clinical and/or patient preferences, as well as choice of definitions, data sources and denominator, can make interpretation of amputation data difficult [10, 11]. Prevalence estimates are also affected by duration of disease. A more comprehensive evaluation of DRLECs, with examination of incidence rates, can improve the understanding of true differences in risk between population groups.

The natural history of type 2 diabetes appears to be different in Asians, who present at relatively younger age, have lower BMI at onset, and have early beta cell dysfunction along with greater insulin resistance and higher risk for renal complications, compared with Western populations [12–15]. However, the designation ‘Asian’ masks the ethnic diversity within Asia. Distinct ethnic groups in Asia include East Asians (indigenous to China, the Korean peninsula and Japan), South Asians (indigenous to the Indian sub-continent) and South-East Asians (indigenous to the region east of India and south of China). Substantial heterogeneity exists between these groups in relation to diabetes pathophysiology, with South Asians showing greater insulin resistance even at low BMI and East Asians showing limited insulin secretory capacity [16–18]. There are also differences in the predisposition to complications, with South Asians more likely to have cardiovascular complications and East Asians more likely to have renal complications [19, 20]. In relation to lower limb complications, South Asians appear to have lower risk of revascularisation and amputation compared with Europeans [21]. However, there is limited data from Asia on the risk and progression from diabetes to these complications, thus far precluding a detailed evaluation of the differences in the predisposition to lower limb complications among the ethnic groups in Asia. Therefore, we investigated the epidemiology of lower limb complications in a multi-ethnic Asian population, using a cohort of individuals with incident type 2 diabetes to ascertain the incidence, time to event and risk factors of DRLECs and amputations.

## Methods

### Participants and data sources

The population in Singapore comprises Chinese, Malays, Indians and other ethnic groups, in decreasing order of proportion. Of these, Malays are indigenous to Singapore while the rest have migrated here over the last two centuries. This multi-ethnic composition offers a unique opportunity to compare South-East Asians (Malays), East Asians (Chinese) and South Asians (Indians) within a common setting.

For this study, we used data from the National Healthcare Group (NHG) Chronic Disease Management System (CDMS). The NHG is one of the three integrated public healthcare clusters in Singapore, serving the central region of the country [22]. The CDMS was developed in 2007 to electronically capture administrative and clinical (diagnosis, laboratory and pharmacy) data from all NHG institutions for patients with chronic conditions [23]. As an electronic registry, the CDMS scans attendances across all NHG institutions on a daily basis to identify new patients with diabetes. Patients are labelled as having diabetes if they meet one of the following criteria: (1) diagnosis codes of 250.0–250.9 (ICD-9-CM; http://www.icd9data.com/2007/Volume1/default.htm) and E10-E14 (ICD-10-CM; http://apps.who.int/classifications/icd10/browse/2016/en); (2) prescription of glucose-lowering medication; and (3) laboratory results in the diabetic range (2 h blood glucose level of ≥11.1 mmol/l or fasting plasma glucose of ≥7.0 mmol/l) [24]. Date of diagnosis is defined as the first date that one of these criteria is met. For all patients with diabetes, the CDMS automatically pulls in data from the institutional electronic medical records, on demographic characteristics, disease profile, comorbidities and clinical and laboratory assessments.

We included for analysis all people who were first diagnosed with type 2 diabetes between 1 January 2007 and 31 December 2016, were aged between 16 and 100 years and who had had no foot problem or amputation previously recorded. The age range was chosen to minimise the erroneous inclusion of individuals with type 1 diabetes, and wrong dates of birth. Individuals were followed up from diagnosis of diabetes until 31 December 2017 or date of death, whichever was earlier. Only the first instances of DRLEC and amputation, as indicated by the earliest date of diagnosis or procedure, were analysed. Since we could not ascertain which limb was affected, lower limb complications were considered to occur in order of severity. Individuals with any DRLEC before the first visit for diabetes were excluded. DRLEC diagnoses after amputation, and amputation without a prior or concurrent DRLEC, were also excluded. Data from a total of 156,593 individuals were included for analysis (see Electronic supplementary material, Fig. 1).

To ensure completeness of data capture for all variables of interest, each individual was also linked to the administrative datasets of the Ministry of Health (MOH). The administrative datasets are comprehensive in their coverage of visits and hospitalisations to public healthcare institutions. They also capture all hospitalisations and day surgeries in private hospitals, as well as selected outpatient visits that are covered under MediSave (the national medical savings account programme), MediShield (the national health insurance programme) and the Community Health Assistance Scheme (a scheme for healthcare subsidies to lower- and middle-income households). Data on deaths were obtained through linkage with the Singapore Registry of Births and Deaths.

This study was approved by the NHG Ethics Review Board (Domain-Specific Review Board).

### Outcomes of interest

#### DRLECs

DRLECs included ulceration, infection, symptomatic peripheral angiopathy, and gangrene with or without angiopathy; and were defined using the ICD-9-CM and ICD-10 codes listed in ESM Table 1. Only the first diagnosis of DRLEC in any individual was included in the analysis.

#### Amputations

Amputations were identified using the MOH Table of Surgical Procedures (ESM Table 2). We excluded traumatic and tumour-related amputations, hence all amputations identified were assumed to be related to diabetes. Only the first ever amputation event was used for analysis.

### Other variables of interest

#### Demographic variables

Demographic variables obtained from the CDMS included date of birth, sex and ethnic group (Chinese, Malay, Indian and Other). Age was calculated as age on the date of diagnosis, and was categorised into groups (<50, 50–59, 60–69, 70–79 and ≥80 years).

#### Mortality

Mortality was defined based on date of death available through linkage with the Singapore Registry of Births and Deaths.

#### Comorbidities

Comorbidities (nephropathy, heart disease, stroke, retinopathy, peripheral vascular disease, neuropathy) were identified using the respective ICD codes in the datasets. These codes are typically assigned by the treating medical professional. The presence of a specific comorbidity was defined as any record of diagnosis before the first visit for diabetes (or first DRLEC) and up to 6 months after.

#### Biochemical and physiological variables

Data on HbA_1c_, lipids, eGFR, mean arterial pressure and BMI were obtained from the CDMS or linked datasets. The closest values within 12 months from the date of first visit for diabetes (or first DRLEC) were used for analysis. Smoking status was categorised based on recorded status throughout the study period.

### Statistical analysis

#### Calculating the incidence of DRLEC and amputation

Cumulative incidence of first DRLEC (or first amputation) was calculated by using the total number of new cases of DRLEC (or amputation) as numerator and the total number of individuals with diabetes (or DRLEC) as denominator. This was averaged over the number of observation years to generate the annual average cumulative incidence. The crude incidence rate of first DRLEC was calculated by using the total DRLEC-free period in person-years as the denominator. For first amputation, the crude incidence rate was calculated by using the total number of first amputations as numerator and the total amputation-free period in those with first DRLEC in person-years as the denominator. Differences in incidence between age, sex and ethnicity categories were analysed using the two-sided Fisher’s exact test for cumulative incidence, and Poisson regression for incidence rate. Age- and sex-standardised incidence rates for first DRLEC and first amputation were calculated using the 2013 national population with diabetes, obtained from the MOH administrative datasets, as the standard population. CIs for the standardised rates were calculated using the Dobson method [25].

#### Calculating time to progression

Time differences between the date of first visit for diabetes and the date of each event (first DRLEC, first amputation) were calculated to construct a progression timeline of lower limb complications in individuals who experienced progression. Time intervals were reported as median and IQR.

#### Risk factors for DRLEC and amputation

Risk factors (demographic, comorbidity, biochemical and physiological variables) for time to first DRLEC and first amputation were analysed using Cox proportional hazard regression [26], allowing for at least 1 year of follow-up. Complete case analysis was undertaken. A separate category of ‘missing’ was created for each variable with missing data at baseline, to be able to include these participants in the analysis. For analysis of risk factors for the first DRLEC, after excluding those with any events within 1 year (*n* = 10,435), 146,158 individuals with diabetes were included in the final regression model. Of these, 14,051 individuals developed DRLEC. After excluding 1603 individuals experiencing events within 1 year of the DRLEC, 12,448 individuals with DRLECs were included in the final regression model for amputation. All variables of interest were entered into the model. Proportional hazard assumption was assessed using Schoenfeld’s residuals test. No variable violated the proportional hazards assumption.

Data management and statistical analyses were performed using R software version 3.5.0 (R Core Team, 2018; available from https://cran.r-project.org/bin/windows/base/) and STATA 16-MP (StataCorp LP, College Station, TX, USA, 2015).

## Results

A total of 156,593 individuals with incident type 2 diabetes were available for analysis in the CDMS, with a median follow-up duration of 7.0 years (IQR 4.4–9.2). Of these, 135,849 did not develop any lower limb complication, 19,536 developed DRLECs only, and 1208 developed DRLECs that progressed to amputation (Table 1). The groups with DRLECs and amputation had a greater proportion of men, non-Chinese ethnic groups, comorbid conditions except for stroke and poorer control or unknown values of biochemical variables at baseline. Ulceration and/or infection were the most common among the DRLECs recorded, comprising 95.7% of all DRLECs in those without amputation, and 84.0% in those with amputation.

**Table 1:** Characteristics of the population with incident type 2 diabetes in the NHG CDMS

Variables	No DRLEC (*n* = 135,849)	With DRLEC only (*n* = 19,536)	With amputation^a^ (*n* = 1208)	*p* value
Age group				<0.001
<50 years	34,554 (25.4)	4838 (24.8)	315 (26.1)	
50–59 years	41,266 (30.4)	5368 (27.5)	404 (33.4)	
60–69 years	35,194 (25.9)	4705 (24.1)	295 (24.4)	
70–79 years	18,040 (13.3)	3181 (16.3)	154 (12.8)	
≥80 years	6795 (5.0)	1444 (7.4)	40 (3.3)	
Male sex	72,656 (53.5)	11,059 (56.6)	802 (66.4)	<0.001
Ethnicity				<0.001
Chinese	88,567 (65.2)	12,403 (63.5)	685 (56.7)	
Malay	17,303 (12.7)	2540 (13.0)	110 (9.1)	
Indian	17,694 (13.0)	3188 (16.3)	300 (24.8)	
Other	12,285 (9.0)	1405 (7.2)	113 (9.4)	
Category of DRLEC				<0.001
Ulceration/infection	–	18,693 (95.7)	1015 (84.0)	
Symptomatic peripheral angiopathy	–	529 (2.7)	41 (3.4)	
Angiopathy with gangrene	–	125 (0.6)	36 (3.0)	
Gangrene	–	189 (1.0)	116 (9.6)	
Presence of chronic comorbidities at DM diagnosis				
Peripheral vascular disease	4396 (3.2)	1041 (5.3)	340 (28.2)	<0.001
Nephropathy	18,030 (13.3)	3271 (16.7)	232 (19.2)	<0.001
Heart disease	27,382 (20.2)	4550 (23.3)	310 (25.7)	<0.001
Stroke	12,675 (9.3)	2123 (10.9)	107 (8.9)	<0.001
Retinopathy and maculopathy	4991 (3.7)	999 (5.1)	201 (16.6)	<0.001
Neuropathy	2810 (2.1)	552 (2.8)	71 (5.9)	<0.001
HbA_1c_				<0.001
<53 mmol/mol (<7%)	47,500 (35.0)	5343 (27.4)	103 (8.5)	
≥53 mmol/mol (≥7%)	53,527 (39.4)	8393 (42.9)	710 (58.8)	
Missing	34,822 (25.6)	5810 (29.7)	395 (32.7)	
eGFR				<0.001
≥60 ml min^−1^ [1.73 m]^−2^	89,899 (66.2)	11,372 (58.2)	597 (49.4)	
<60 ml min^−1^ [1.73 m]^−2^	12,928 (9.5)	2432 (12.5)	176 (14.6)	
Missing	33,022 (24.3)	5732 (29.3)	435 (36.0)	
LDL-cholesterol				<0.001
<2.6 mmol/l	30,314 (22.3)	3918 (20.1)	221 (18.3)	
≥2.6 mmol/l	65,859 (48.5)	8841 (45.3)	438 (36.3)	
Missing	39,676 (29.2)	6777 (34.7)	549 (45.5)	
Mean arterial pressure				<0.001
<100 mmHg	73,541 (54.1)	9811 (50.2)	446 (36.9)	
≥100 mmHg	29,981 (22.1)	4326 (22.1)	242 (20.0)	
Missing	32,327 (23.8)	5399 (27.6)	520 (43.1)	
BMI				<0.001
≤27.5 kg/m^2^	54,173 (39.9)	7112 (36.4)	409 (33.9)	
>27.5 kg/m^2^	37,859 (27.9)	5246 (26.9)	138 (11.4)	
Missing	43,817 (32.3)	7178 (36.7)	661 (54.7)	
Smoking status				<0.001
Non-smoker	74,417 (54.8)	11,148 (57.1)	524 (43.4)	
Ever smoker	15,340 (11.3)	2894 (14.8)	221 (18.3)	
Missing	46,092 (33.9)	5494 (28.1)	463 (38.3)	

Data are presented as *n* (%) unless otherwise stated

Total observations: *n* = 156,593. Biochemical variables are taken as the closest value within 1 year from type 2 diabetes diagnosis; comorbidities were taken at any record before type 2 diabetes diagnosis up to 6 months after diagnosis

^a^Only for those who were diagnosed with DRLEC and then progressed to any amputation, excluding those without any DRLEC diagnosis recorded who underwent amputations (*n* = 185)

*p* values are from χ^2^ tests, comparing differences across the three groups

### Rates of progression to first DRLEC and first amputation

The crude incidence of first DRLEC was 25.34/1000 person-years among individuals with diabetes (Table 2). The crude incidence of first DRLEC was higher in men than in women (27.42 vs 23.01/1000 person-years, *p* < 0.001), lowest in those aged 50–59 years (22.72/1000 person-years) and highest in those aged 80 years and above (47.13/1000 person-years). The overall age- and sex-standardised incidence rate of first DRLEC was 28.29/1000 person-years among individuals with diabetes. When comparing ethnic groups, both crude and age- and sex-standardised incidence rates were highest in Malays (crude 32.75/1000 person-years, *p* < 0.001; standardised 36.09/1000 person-years).

**Table 2:** Incidence of first DRLEC and first lower extremity amputation, 2007–2017

Indicator	*n*	First DRLEC in people with type 2 diabetes^a^					First amputation in people with DRLEC^b^				
		*n*	Crude	*p* value	Standardised^c^ (95% CI)	Rate ratio (95% CI)	*n*	Crude	*p* value	Standardised^c^ (95% CI)	Rate ratio (95% CI)
Overall		20,744					1208				
Cumulative incidence, annual average (% per year)			1.20	–	–	**–**		0.53	–	**–**	–
Cumulative incidence, annual average (/100,000 per year)			1204.28		–			529.40		**–**	
Incidence rate, total (/1000 person-years)			25.34		28.29 (27.82, 28.75)			7.28		8.18 (7.69, 8.70)	
Cumulative incidence, annual average (% per year)											
Sex											
Female	72,076	8883	1.12	<0.001	**–**	**–**	406	0.42	<0.001	**–**	–
Male	84,517	11,861	1.28		**–**		802	0.61		**–**	
Ethnicity											
Chinese	101,655	13,088	1.17	<0.001	**–**	**–**	685	0.48	<0.001	**–**	–
Malay	21,182	3488	1.50		**–**		300	0.78		**–**	
Indian	19,953	2650	1.21		**–**		110	0.38		**–**	
Other	13,803	1518	1.00		**–**		113	0.68		**–**	
Age											
< 50 years	39,707	5153	1.18	<0.001	**–**	**–**	315	0.56	<0.001	**–**	–
50–59 years	47,038	5772	1.11		**–**		404	0.64		**–**	
60–69 years	40,194	5000	1.13		**–**		295	0.54		**–**	
70–79 years	21,375	3335	1.42		**–**		154	0.42		**–**	
≥ 80 years	8279	1484	1.63		**–**		40	0.25		**–**	
Incidence rate, total (/1000 person-years)											
Sex											
Female	72,076	8883	23.01	Ref	**–**	**–**	406	6.74	Ref	**–**	**–**
Male	84,517	11,861	27.42	<0.001	**–**	**–**	802	10.24	<0.001	**–**	**–**
Ethnicity											
Chinese	101,655	13,088	24.51	Ref	27.47 (26.94, 28.00)	Ref	685	7.84	Ref	7.36 (6.80, 7.95)	Ref
Malay	21,182	3488	32.75	<0.001	36.09 (34.31, 37.91)	1.31 (1.23, 1.41)	300	13.06	<0.001	12.96 (11.05, 15.04)	1.76 (1.39, 2.21)
Indian	19,953	2650	24.88	0.145	27.75 (26.05, 29.50)	1.01 (0.93, 1.10)	110	5.93	0.051	5.42 (4.12, 6.92)	0.74 (0.52,1.02)
Other	13,803	1518	21.00	<0.001	22.06 (20.53, 23.64)	0.80 (0.73, 0.88)	113	11.59	0.055	12.98 (10.15, 16.23)	1.76 (1.28,2.39)
Age											
<50 years	39,707	5153	23.64	Ref	–	**–**	315	8.59	Ref	**–**	**–**
50–59 years	47,038	5772	22.72	0.003	–	**–**	404	10.25	0.291	**–**	**–**
60–69 years	40,194	5000	24.09	0.033	–	**–**	295	8.98	0.337	**–**	**–**
70–79 years	21,375	3335	31.15	<0.001	–	**–**	154	7.11	0.327	**–**	**–**
≥80 years	8279	1484	47.13	<0.001	–	**–**	40	5.07	0.003	**–**	**–**

Cumulative incidence = annual average = (total number of new cases / total number of individuals) / number of observation years

Incidence rate = total number of new cases / sum of person-years for event-free period

^a^At-risk population: individuals with type 2 diabetes with no previous DRLEC (*n* = 156,593)

^b^At-risk population: individuals with type 2 diabetes who have had DRLEC (*n* = 20,744)

^c^Age- and sex-standardised to 2013 Singapore population with diabetes

*p* value from two-sided Fisher’s exact test for cumulative incidence, comparing differences across groups; from Poisson regression for incidence rate

The crude incidence of first amputation was 7.28/1000 person-years among individuals with DRLEC, while the age- and sex-standardised rate was 8.18/1000 person-years (Table 2). Again, incidence was higher in men than in women (10.24 vs 6.74/1000 person-years, *p* < 0.001) and highest among Malays (crude 13.06/1000 person-years, *p* < 0.001; standardised 12.96/1000 person-years). However, amputation incidence did not vary significantly by age, except in the oldest age group.

### Time to progression from first visit for diabetes to first DRLEC and first amputation

**Fig. 1:**
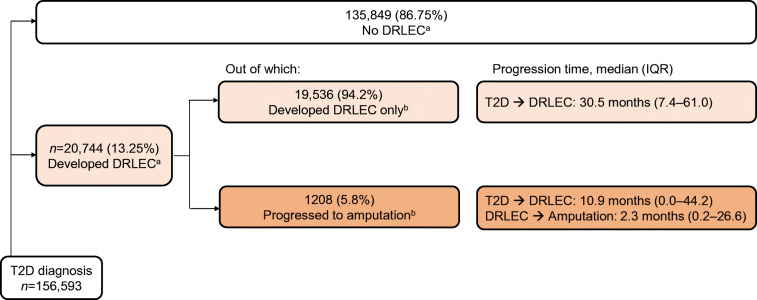
Time to progression for lower limb complications among individuals with incident type 2 diabetes, 2007–2017. ^a^Out of all individuals in the CDMS with T2D diagnosis; ^b^out of individuals who developed DRLEC. T2D, type 2 diabetes mellitus

The median time interval between the first public sector diagnosis of diabetes and the first occurrence of DRLEC was 30.5 months (IQR 7.4–61.0) in individuals who did not progress after DRLEC, and 10.9 months (IQR 0.0–44.2) for those with subsequent amputation (Fig. 1). One-quarter of individuals undergoing an amputation procedure were diagnosed with a DRLEC at their first visit for diabetes. The median time to progression from DRLEC to first amputation was 2.3 months (IQR 0.2–26.6). Progression times to DRLEC appeared shorter for men, Malays and certain age groups, with slightly longer time for progression from DRLEC to first amputation (ESM Table 3).

### Risk factors for progression to first DRLEC and first amputation

Risk factors for progression to first DRLEC included male sex (HR 1.10 [95% CI 1.06, 1.14], *p* < 0.001), Malay ethnicity (HR 1.29 [95% CI 1.23, 1.35], *p* < 0.001) and Indian ethnicity (HR 1.07 [95% CI 1.01, 1.13], *p* = 0.014) (Table 3). Hazards for DRLEC were also increased with increasing age, and in the presence of nephropathy, heart disease, stroke, retinopathy and neuropathy at diagnosis. Individuals with HbA_1c_ ≥ 53 mmol/mol (≥7%) or missing HbA_1c_, with eGFR < 60 ml min^−1^ [1.73 m]^−2^ or missing eGFR, and BMI > 27.5 kg/m^2^ or missing BMI at the time of diagnosis also had higher hazards of DRLEC, as did ever smokers and those with missing data on LDL-cholesterol. Individuals with missing data on mean arterial pressure and smoking status had lower hazards of DRLEC.

**Table 3:** Factors associated with risk of developing first DRLEC and first amputation

Variable	First DRLEC after type 2 diabetes^a^			First amputation after DRLEC^b^		
	Adj. HR^c^	95% CI	*p* value	Adj. HR^c^	95% CI	*p* value
Age at first visit for type 2 diabetes			<0.001			0.080
<50 years	Ref			Ref		
50–59 years	1.02	0.97, 1.07	0.484	0.96	0.69, 1.34	0.832
60–69 years	1.19	1.14, 1.26	<0.001	0.63	0.41, 0.97	0.036
70–79 years	1.64	1.55, 1.74	<0.001	0.56	0.33, 0.97	0.037
≥80 years	2.42	2.24, 2.62	<0.001	0.63	0.29, 1.37	0.242
Male sex	1.10	1.06, 1.14	<0.001	1.44	1.06, 1.95	0.018
Ethnicity			<0.001			0.003
Chinese	Ref			Ref		
Malay	1.29	1.23, 1.35	<0.001	1.27	0.90, 1.80	0.172
Indian	1.07	1.01, 1.13	0.014	0.49	0.29, 0.83	0.007
Other	0.83	0.77, 0.89	<0.001	1.44	0.90, 2.28	0.126
Chronic comorbidities at first visit for type 2 diabetes						
Nephropathy	1.25	1.19, 1.32	<0.001	1.20	0.87, 1.66	0.263
Heart disease	1.17	1.12, 1.22	<0.001	1.15	0.84, 1.56	0.382
Stroke	1.24	1.17, 1.31	<0.001	0.87	0.57, 1.31	0.499
Retinopathy	1.44	1.33, 1.55	<0.001	3.66	2.69, 4.98	<0.001
Peripheral vascular disease	0.96	0.86, 1.07	0.457	2.33	1.65, 3.28	<0.001
Neuropathy	1.22	1.09, 1.37	<0.001	1.23	0.79, 1.92	0.367
HbA_1c_						
<53 mmol/mol (<7%)	Ref					
≥53 mmol/mol (≥7%)	1.28	1.23, 1.34	<0.001	2.24	1.46, 3.43	<0.001
Missing	1.28	1.21, 1.36	<0.001	1.33	0.76, 2.33	0.321
eGFR						
≥60 ml min^−1^ [1.73 m]^−2^	Ref					
<60 ml min^−1^ [1.73 m]^−2^	1.12	1.05, 1.20	<0.001	1.15	0.75, 1.75	0.517
Missing	1.08	1.03, 1.13	0.002	1.10	0.71, 1.69	0.668
LDL-cholesterol						
<2.6 mmol/l	Ref					
≥2.6 mmol/l	1.03	0.99, 1.08	0.160	1.68	1.14, 2.49	0.009
Missing	1.21	1.14, 1.30	<0.001	2.40	1.50, 3.85	<0.001
Mean arterial pressure						
<100 mmHg	Ref					
≥100 mmHg	1.02	0.98, 1.07	0.299	0.93	0.65, 1.35	0.715
Missing	0.87	0.82, 0.92	<0.001	0.98	0.65, 1.47	0.913
BMI						
≤27.5 kg/m^2^	Ref					
>27.5 kg/m^2^	1.15	1.10, 1.20	<0.001	0.93	0.64, 1.34	0.692
Missing	1.20	1.14, 1.27	<0.001	1.70	1.15, 2.51	0.008
Smoking status						
Non-smoker	Ref					
Ever smoker^d^	1.29	1.23, 1.36	<0.001	1.46	1.00, 2.12	0.050
Missing	0.60	0.58, 0.63	<0.001	1.10	0.79, 1.55	0.569

^a^In population with incident type 2 diabetes (*n* = 156,593): excluding those with any event within 1 year, *n* = 10,435; available for analysis, *n* = 146,158. Biochemical variables are taken as closest value within 1 year from diabetes diagnosis and comorbidities are taken at any record before diabetes and up to 6 months after diabetes diagnosis

^b^In population with first DRLEC (*n* = 14,051): excluding those with any event within 1 year, *n* = 1603; available for analysis, *n* = 12,448. Biochemical variables are taken as closest value within 1 year from DRLEC and comorbidities are taken at any record before DRLEC and up to 6 months after DRLEC

^c^Cox proportional hazard analysis, all variables entered into the model

^d^Ever smoker was taken as any record of smoking throughout the study period

Risk factors for progression to amputation included male sex (HR 1.44 [95% CI 1.06, 1.95], *p* = 0.018), presence of retinopathy (HR 3.66 [95% CI 2.69, 4.98], *p* < 0.001), presence of peripheral vascular disease (HR 2.33 [95% CI 1.65, 3.28], *p* < 0.001), HbA_1c_ ≥ 53 mmol/mol (≥7%) (HR 2.24 [95% CI 1.46, 3.43], *p* < 0.001), LDL-cholesterol ≥2.6 mmol/l (HR 1.68 [95% CI 1.14, 2.49], *p* = 0.009) or missing LDL-cholesterol (HR 2.4 [95% CI 1.5, 3.85], *p* < 0.001) and missing BMI (HR 1.7 [95% CI 1.15, 2.51], *p* = 0.008). Indian ethnicity (HR 0.49 [95% CI 0.29, 0.83], *p* = 0.007) was associated with a lower hazard of amputation.

## Discussion

We found that in this population with incident type 2 diabetes, the time intervals from diabetes diagnosis to DRLEC, and from DRLEC to amputation, were relatively short, with the incidence of first ever DRLEC being 28.29/1000 person-years with diabetes and incidence of first ever amputation being 8.18/1000 person-years with DRLEC. Key risk factors for DRLEC included older age, male sex and non-Chinese ethnicity as well as presence of comorbidities and poorer biochemical profiles at diabetes diagnosis. Risk of amputation was mainly associated with poorer biochemical profiles and presence of comorbidities.

We found significant ethnic differences in the incidence of both DRLECs and amputation in our population. South East Asian Malays had much higher incidence of both DRLECs and amputations compared with East Asian Chinese, while there were no differences between South Asian Indians and Chinese. The higher incidence in Malays could be due to differences in risk factor levels between ethnic groups. In Singapore, the prevalence of smoking is highest among Malays of male sex [27]. Malay individuals with diabetes have also been reported to have higher mean BMI [28], a greater proportion of peripheral vascular disease [29], poorer glycaemic control [30], and a greater risk of cardiovascular and chronic kidney disease [31], compared with individuals of Chinese ethnicity. Indeed, there was no difference between Malays and Chinese in hazards of amputation among those with DRLEC after adjustment for disease-related variables and comorbid conditions, though the differences in DRLEC incidence persisted. On the other hand, Indians had a higher hazard for DRLEC compared with Chinese after adjustment, suggesting that lower comorbidity burden in Indians masks differences in DRLEC risk. It is possible that the aetiopathological processes for diabetes and complications differ among these ethnic groups. It has been previously reported that South Asians have a lower risk of peripheral arterial disease compared with Europeans [32], due to greater predilection for coronary rather than peripheral arterial atherosclerosis [21]. Similarly, greater microvascular supply to the skin has been put forth as a potential explanation for the lower risk of peripheral neuropathy in South Asians [33]. However, the risk of peripheral neuropathy and peripheral arterial disease in Malays and Chinese in comparison with other ethnic groups has not been studied. While we were unable to report the incidence of peripheral neuropathy and peripheral arterial disease in our study due to their poor capture in the administrative datasets, future studies should examine ethnic differences in the incidence of these precursor events in our population in relation to the risk of DRLEC.

Differential utilisation of health services due to differences in healthcare beliefs, health literacy, language barriers and other socioeconomic factors may also contribute to the ethnic differences observed in DRLEC incidence. While some of these factors have been evaluated in relation to other conditions [34–37], little is known about their relation to ethnic differences in health service utilisation for diabetes and foot care. More work is therefore needed to unravel the aetiopathological as well as sociocultural mechanisms driving DRLEC among the different ethnic groups studied here.

Half of the individuals who developed a DRLEC did so within 3 years of diabetes diagnosis, while a one-quarter of those progressing to amputation already had a DRLEC at their first diabetes visit. Given that the risk of foot ulceration increases with increasing duration of diabetes [38], these findings suggest that individuals with diabetes present to the public health system late, with limited time in which health professionals can intervene and influence the natural history of DRLEC and subsequent amputation. This could be due to delayed diagnosis of diabetes, or management outside the public sector until complications develop. Healthcare utilisation and access issues could, therefore, be driving the apparently short progression times. It is known that healthcare utilisation, especially of preventive services, is determined by sociocultural factors [39]. Individuals may delay seeking care for diabetes until long after disease onset and may prioritise work or family over health, due to considerations of time, cost and limited access to care beyond working hours. These findings should serve as an impetus for increasing the penetration of the national screening programme, as well as supporting the private primary care sector to enable more effective diabetes management early in the course of the disease.

To the best of our knowledge, this is the first study to report the incidence of lower extremity complications, especially non-amputation complications, comprehensively over a decade. This allows more accurate estimation of the burden and risk of these complications in individuals with type 2 diabetes. However, this does make it difficult to compare our results with previously published literature, since most studies have reported the incidence of diabetic foot ulcers only. In addition, we could not find any study in individuals with incident diabetes. In populations with prevalent diabetes, the reported incidence of first diabetic foot ulcer was 6.1 per 1000 patients in 2013–2017 in the UK [40] and 2.1 per 1000 person-years in Japan [41], while new ulcer incidence was 0.34% per year in the Netherlands [42]. In terms of incidence of amputation in individuals with DRLECs, most previous reports have had short follow-up times of 2 or 3 years, which again limits comparability with our study. The annual incidence of amputation in those with diabetic foot ulcers and/or peripheral arterial disease was reported to be 1.1% in 2016 in South Korea [43]. Cumulative incidences of 10% in 2 years in Scotland [44] and 5.8% in 3 years in Portugal [45] have been reported in individuals with diabetes and high-risk foot. While not directly comparable with our results, these data cumulatively suggest a relatively higher incidence of DRLEC, but not amputation, in our population. Our current findings, therefore, emphasise the need to place focus early in the natural history of diabetes, before the development of DRLEC, in order to reduce the burden of amputations.

One of the main strengths of our study is the investigation of the incidence of DRLECs as a two-step process, from diabetes diagnosis to DRLECs, and from DRLECs to first amputation. Ours is one of the few studies to look at a comprehensive set of DRLECs, rather than individual complications. This has enabled us to present a complete picture of the burden of these complications in our population. The linkage with the national administrative dataset and the death registry has minimised loss to follow-up through the nationwide capture of healthcare interactions and deaths, with standardised coding across healthcare institutions for disease diagnoses and amputation procedures. Another key strength is the examination of ethnic differences in the risk of lower extremity complications within the context of a common health system, thus eliminating differences due to methodology or healthcare provision. The ethnic differences we report can therefore be clearly attributed to disparities in healthcare utilisation and/or differential risk. Our findings not only shed light on DRLECs in the Asian context but also raise interesting questions for future researchers to delve into.

This study also has some limitations. The analysis was limited to individuals with type 2 diabetes, since the proportion of individuals identified as having type 1 diabetes in the CDMS was extremely small, with very low numbers of DRLEC and amputation events. Diagnosis of diabetes was based on data from public healthcare institutions. Individuals with diabetes diagnosis outside the public sector may have presented an artificially short time from first diabetes visit to DRLEC. This may increase the uncertainty around our estimates. However, it does not affect the validity of our findings that individuals present late to the public health system. All DRLEC events were combined into one single DRLEC variable, due to small numbers for subgroup analysis for the individual DRLEC subtypes. This may mask differences in progression to amputation between the DRLEC subtypes. Limb salvage efforts such as debridement and revascularisation procedures were not examined in relation to risk of amputation due to data limitations. The capture of precursor events such as peripheral neuropathy and peripheral arterial disease is poor in the administrative data, both at national and regional levels. Therefore, we were unable to map progression from diabetes to these conditions in the pathway to DRLEC.

In conclusion, this comprehensive evaluation of the epidemiology of lower limb complications in a multi-ethnic Asian population has revealed important ethnic differences, with Malays having the highest incidence of both DRLEC and amputations, and greatest risk of progression to DRLEC after adjusting for potential confounders. Differences in the pathophysiology of diabetes and its complications, burden of comorbid conditions and health service utilisation may be potential reasons for the observed ethnic differences. Greater research efforts are needed to understand the aetiopathological and sociocultural processes that contribute to the higher risk of lower extremity complications among these ethnic groups. At the same time, greater and sustained focus on improving diabetes care in general, and diabetic foot care in particular, will be needed to reduce the transition to DRLEC in this multi-ethnic population.

## Supplementary Information

ESM (PDF 347 kb)

## Data Availability

The data used in this study are from the MOH Singapore administrative datasets and the NHG CDMS and are not publicly available. Requests for onsite access to the anonymised data for research may be made to the respective organisations.

## Abbreviations

CDMS: Chronic Disease Management System
DRLEC: Diabetes-related lower extremity complication
MOH: Ministry of Health
NHG: National Healthcare Group
