# Supplementary material for: Diabetes-related lower extremity complications in a multi-ethnic Asian population: a 10 year observational study in Singapore
Source: Diabetologia. 2021 Apr 22;64(7):1538–49. doi: 10.1007/s00125-021-05441-3 (PMC8187215; doi:10.1007/s00125-021-05441-3)
Supplement: Supplementary file 1 — (PDF 347 kb) [file 125_2021_5441_MOESM1_ESM.pdf]

ELECTRONIC SUPPLEMENTARY MATERIAL (ESM)

ESM Table 1 List of diagnosis codes for diabetes-related lower extremity complications

Diagnosis Catalogue	Diagnosis Code	Description
ICD9CM	250.7	Diabetes mellitus, Diabetes with peripheral circulatory disorders
ICD9CM	440.2	Atherosclerosis, Of native arteries of the extremities
ICD10	I70.20	Atherosclerosis Of Arteries Of Extremities, unspecified
ICD9CM	440.21	Atherosclerosis of arteries of extremities with claudication
ICD9CM	440.22	Atherosclerosis of arteries of extremities with rest pain
ICD9CM	440.23	Atherosclerosis of arteries of extremities with ulceration
ICD9CM	440.24	Atherosclerosis of arteries of extremities with gangrene
ICD10	E11.73	Type 2 diabetes mellitus with peripheral angiopathy, with gangrene
ICD10	E14.52	Unspecified diabetes mellitus with peripheral angiopathy, with gangrene
ICD10	I70.20	Atherosclerosis of arteries of extremities, unspecified
ICD10	I70.23	Atherosclerosis of arteries of extremities with ulceration
ICD10	I70.24	Atherosclerosis of arteries of extremities with gangrene
ICD10	I83.0	Varicose veins of lower extremities with ulcer
ICD10	I83.2	Varicose veins of lower extremities with both ulcer and inflammation
ICD9CM	40	Gas gangrene
ICD9CM	785.4	Gangrene
ICD10	A48.0	Gas gangrene
ICD10	R02	Gangrene, not elsewhere classified
ICD9CM	680.7	Carbuncle of foot
ICD9CM	681	Cellulitis and abscess of finger and toe
ICD9CM	681.1	Cellulitis and abscess of finger and toe, toe
ICD9CM	681.11	Onychia of toe
ICD9CM	682	Other cellulitis and abscess
ICD9CM	682.6	Cellulitis of leg
ICD9CM	682.7	Cellulitis of foot
ICD9CM	682.8	Cellulitis, site NEC
ICD9CM	682.9	Cellulitis NOS
ICD9CM	730.07	Acute osteomyelitis-ankle
ICD9CM	730.17	Chronic osteomyelitis-ankle
ICD9CM	730.27	Osteomyelitis NOS-ankle
ICD10	L03	Cellulitis
ICD10	L03.02	Cellulitis of toe
ICD10	L03.11	Cellulitis of lower limb
ICD10	L03.8	Cellulitis of other sites
ICD10	L03.9	Cellulitis, unspecified
ICD10	M86.17	Other acute osteomyelitis, ankle and foot
ICD10	M86.67	Other chronic osteomyelitis, ankle and foot
ICD9CM	707.1	Chronic ulcer of leg
ICD10	E13.73	Type 2 diabetes mellitus with foot ulcer due to multiple causes

ICD10	L89.9	Decubitus ulcer and pressure area, unspecified
ICD10	L97	Ulcer of lower limb, not elsewhere classified
ICD10	L98.4	Chronic ulcer of skin, not elsewhere classified
ICD10	S91.1	Open wound of toe(s) without damage to nail
ICD10	S91.2	Open wound of toe(s) with damage to nail
ICD10	S91.3	Open wound of other parts of foot

ESM Table 2 List of procedure codes for lower extremity amputations

Surgical Procedure Code	Surgical Procedure Description
SB400T	Toe, Various Lesions, Amputation (Single)
SB708T	Toes, Various Lesions, Ray Amputation (single)
SB707T	Toes, Various Lesions, Ray Amputation (multiple)
SB401T	Toe, Various Lesions, Amputation (Multiple)
SSB400T	Toe, Various Lesions, Amputation
SSB708T	Toes, Various Lesions, Ray Amputation (single)
DSB707T	Toes, Various Lesions, Ray Amputation (multiple)
SSB401T	Toe, Various Lesions, Amputation
DSB708T	Toes, Various Lesions, Ray Amputation (single)
SLB400T	Toe, Various Lesions, Amputation
SSB707T	Toes, Various Lesions, Ray Amputation (multiple)
SB829T	Toes, Various Lesions, Amputation (multiple)
SSB829T	Toes, Various Lesions, Amputation (multiple)
SSB830T	Toes, Various Lesions, Amputation (single)
DSB400T	Toe, Lesions, Amputation (Single)
DSB401T	Toe, Lesions, Amputation (Multiple)
SB830T	Toes, Various Lesions, Amputation (Single)
DSB829T	Toes, Various Lesions, Amputation (multiple)
SLB401T	Toe, Various Lesions, Amputation
DSB830T	Toes, Various Lesions, Amputation (single)
DLB400T	Toe, Lesions, Amputation (Single)
LB401T	Toe, Various Lesions, Amputation (Multiple)
SB010L	Lower Limb, Various Lesions, Amputation
SB809L	Lower Limb, Various Lesions, Amputation
SSB010L	Lower Limb, Various Lesions, Amputation
SSB809L	Lower Limb, Various Lesions, Amputation
SLB010L	Lower Limb, Various Lesions, Amputation
DSB809L	Lower Limb, Various Lesions, Amputation
SB013L	Lower Limb, Various Lesions, Hip Disarticulation
DSB010L	Lower Limb, Various Lesions, Amputation
SSB013L	Lower Limb, Various Lesions, Hip Disarticulation
SSB812L	Lower Limb, Various Lesions, Hip Disarticulation
SSB012L	Lower Limb, Various Lesions, Hindquarter Amputation

ESM Table 3 Progression timelines of diabetes-related lower extremity complications by sex, ethnicity and age group in patients with incident type 2 diabetes mellitus

**Characteristics**	**Progression**	**Number of people, n (%)**	**Time from T2D to DRELC in months, median (IQR)**	**Time from DRLEC to Amputation in months, median (IQR)**
Females (n=72,076)	No DRLEC	63,193 (87.7)		
	With DRLEC	8,883 (12.3)		
	With DRLEC only	8,477 (95.4)	33.9 (9.9-64.5)	-
	Progressed to Amputation	406 (4.6)	13.6 (0.0-51.7)	1.8 (0.2-23.0)
Males (n=84,517)	No DRLEC	72,656 (86.0)		
	With DRLEC	11,861 (14.0)		
	With DRLEC only	11,059 (93.2)	28.0 (5.8-58.1)	-
	Progressed to Amputation	802 (6.8)	10.2 (0.0-41.8)	2.9 (0.2-29.1)
Chinese (n=101,655)	No DRLEC	88,567 (87.1)		
	With DRLEC	13,088 (12.9)		
	With DRLEC only	12,403 (94.8)	32.6 (9.2-63.2)	-
	Progressed to Amputation	685 (5.2)	13.2 (0.0-46.2)	2.1 (0.2-25.6)
Malay (n=21,182)	No DRLEC	17,694 (83.5)		
	With DRLEC	3,488 (16.5)		
	With DRLEC only	3,188 (91.4)	28.6 (6.6-59.0)	-
	Progressed to Amputation	300 (8.6)	8.2 (0.0-46.1)	2.8 (0.2-27.9)
Indian (n=19,953)	No DRLEC	17,303 (86.7)		
	With DRLEC	2,650 (13.3)		
	With DRLEC only	2,540 (95.8)	27.1 (5.4-57.3)	-
	Progressed to Amputation	110 (4.2)	12.8 (0.3-34.2)	2.5 (0.2-24.0)
Others (n=13,803)	No DRLEC	12,285 (89.0)		
	With DRLEC	1,518 (11.0)		
	With DRLEC only	1,405 (92.6)	21.5 (1.2-51.4)	-
	Progressed to Amputation	113 (7.4)	4.3 (0.0-30.2)	1.3 (0.1-31.2)

Age <50 (n=39,707)	No DRLEC	34,554 (87.0)		
	With DRLEC	5,153 (13.0)		
	With DRLEC only	4,838 (93.9)	26.6 (4.7-58.4)	-
	Progressed to Amputation	315 (6.1)	14.1 (0.0-50.9)	9.0 (0.2-40.1)
Age 50-59 (n=47,038)	No DRLEC	41,266 (87.7)		
	With DRLEC	5,772 (12.3)		
	With DRLEC only	5,368 (93.0)	29.8 (6.7-59.6)	-
	Progressed to Amputation	240 (7.0)	8,2 (0.0-37.4)	3.5 (0.2-35.5)
Age 60-69 (n=40,194)	No DRLEC	35,194 (87.6)		
	With DRLEC	5,000 (12.4)		
	With DRLEC only	4,705 (94.1)	32.9 (9.1-63.7)	-
	Progressed to Amputation	295 (5.9)	10.8 (0.0-45.1)	1.0 (0.1-11.5)
Age 70-79 (n=21,375)	No DRLEC	18,040 (84.4)		
	With DRLEC	3,335 (15.6)		
	With DRLEC only	3,181 (95.4)	36.0 (11.6-66.5)	-
	Progressed to Amputation	154 (4.6)	13.3 (0.0-46.8)	1.6 (0.3-18.3)
Age >=80 (n=8,279)	No DRLEC	6,795 (82.1)		
	With DRLEC	1,484 (17.9)		
	With DRLEC only	1,444 (97.3)	27.8 (7.4-52.6)	-
	Progressed to Amputation	40 (2.7)	8.4 (0.1-28.9)	1.7 (0.4-18.7)

DRLEC = diabetes-related lower extremity complication; T2D = type 2 diabetes mellitus

ESM Fig. 1 Flowchart of participants included for analysis of progression from diagnosis of type 2 diabetes to first DRLEC and first amputation, National Healthcare Group Chronic Disease Management Registry

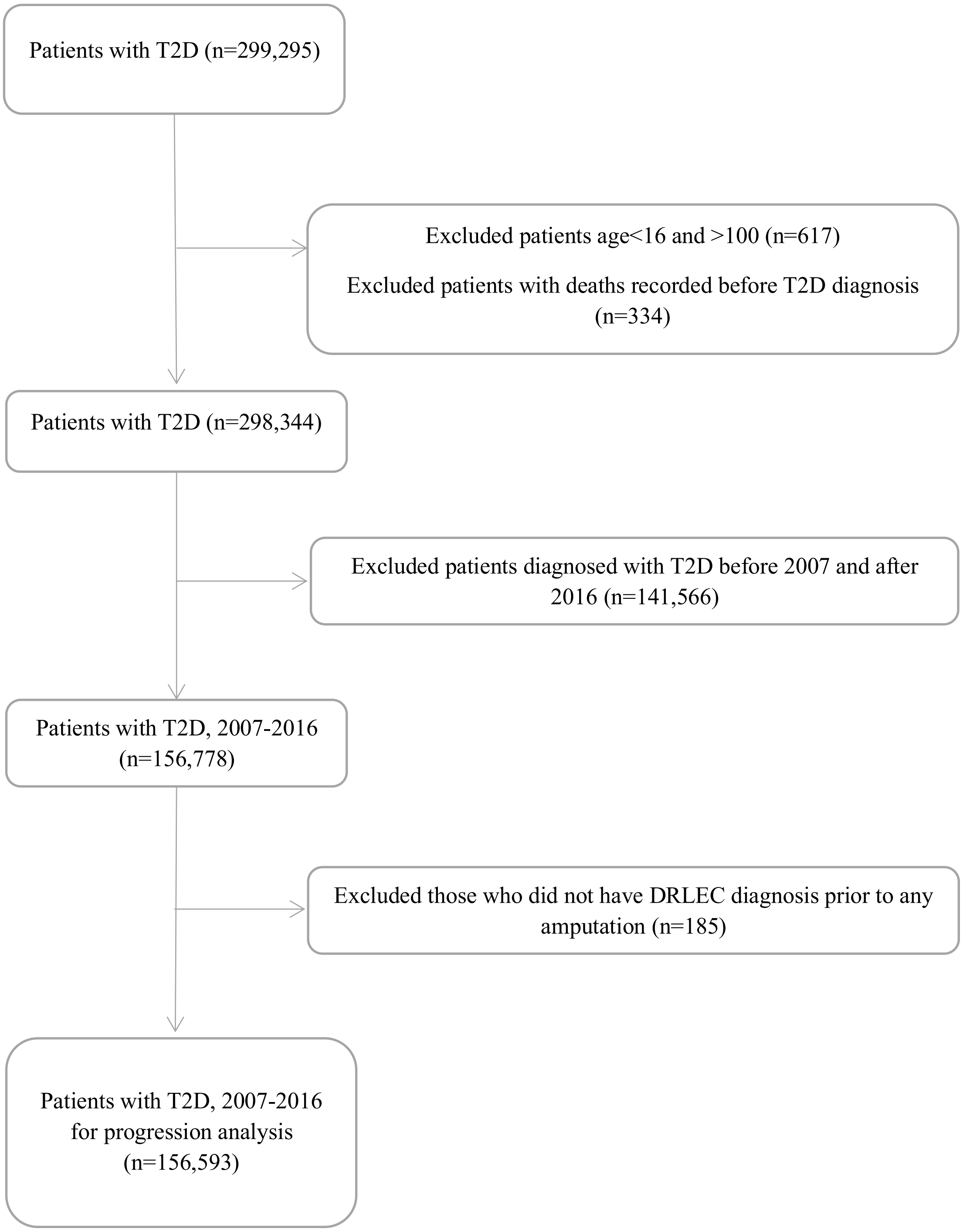

DRLEC = diabetes-related lower extremity complication; T2D = type 2 diabetes mellitus
